# Lateral Thoracic Osteoplastic Rib-Sparing Technique Used for Lateral Spine Surgery: Technical Note

**DOI:** 10.7759/cureus.668

**Published:** 2016-07-05

**Authors:** Marc Moisi, Christian Fisahn, R. Shane Tubbs, Jeni Page, Richard Rice, David Paulson, Noojan Kazemi, David Hanscom, Rod J Oskouian

**Affiliations:** 1 Seattle Science Foundation; 2 Neurological Surgery, Wayne State University; 3 Neurosurgery, Swedish Neuroscience Institute; 4 Department of Trauma Surgery, BG University Hospital Bergmannsheil, Bochum, Germany; 5 Neurosurgery, Seattle Science Foundation; 6 Neurosurgey, Swedish Neuroscience Institute; 7 Neurosurgery, University of Arkansas; 8 Neurosurgery, Complex Spine, Swedish Neuroscience Institute

**Keywords:** rib sparing, thoracic corpectomy, lateral interbody fusion, rib preservation, thoracic lateral interbody fusion, postoperative rib pain

## Abstract

Of patients who have undergone lateral approaches to the thoracic spine, surgical site postoperative pain appears to be greater among those who have undergone transection and removal of a rib segment than those who have not. Therefore, techniques that conserve anatomical position and minimize tissue disruption would theoretically result in less pain and a quicker recovery. Herein, we describe a rib-sparing osteoplastic technique used when rib segments need to be displaced in order to create an unobscured corridor to the operative target. Our approach minimizes soft tissue disruption and restores the anatomical function of the rib. Based on our experience, these patients report less pain, mobilize earlier, and are discharged sooner than those who have had rib segments sacrificed as part of a lateral approach to the spine.

## Introduction

A recent innovative technique in spine surgery for access to disc spaces and vertebral bodies is the lateral approach. As spine surgeons have become more comfortable with this approach to the lumbar spine, the procedure has been adapted for use at thoracic levels. For upper lumbar and thoracic levels, the method often necessitates the removal of a short segment of the rib. However, a common complaint from patients is the exaggerated pain at this rib harvest site. We postulated that this pain was due to focal rib instability termed "a flail segment". Due to this instability, we hypothesized that the stabilization of this segment would result in decreased pain and improved patient satisfaction. Thus, we devised an osteoplastic rib-preserving method to stabilize flail rib segments removed during lateral approaches to the upper lumbar and thoracic spine. The technique was used for four patients. In this small series, the self-reported pain levels were noticeably less and the hospital stays were decreased compared to patients undergoing the same procedure but without the rib-preserving technique. 

## Technical report

### Illustrative cases

**Figure 1:**
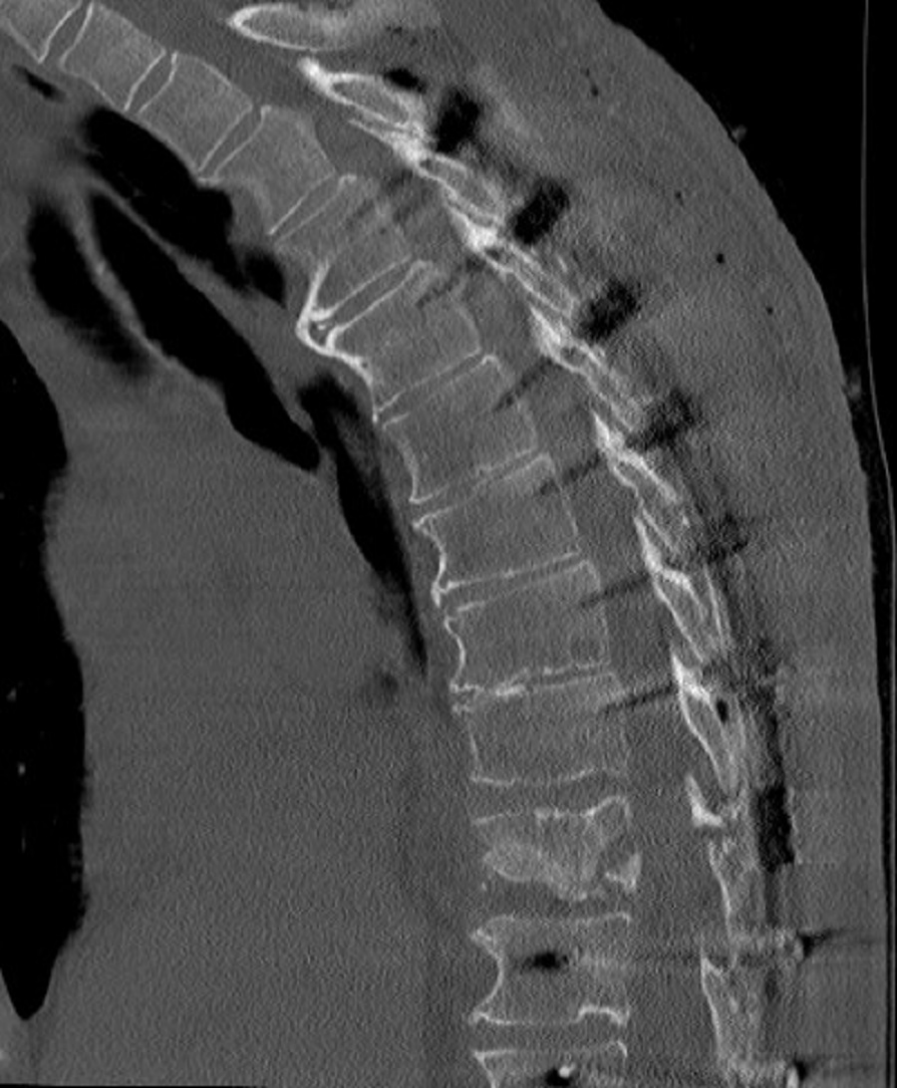
Preoperative sagittal CT demonstrating a T10 burst fracture

**Figure 2:**
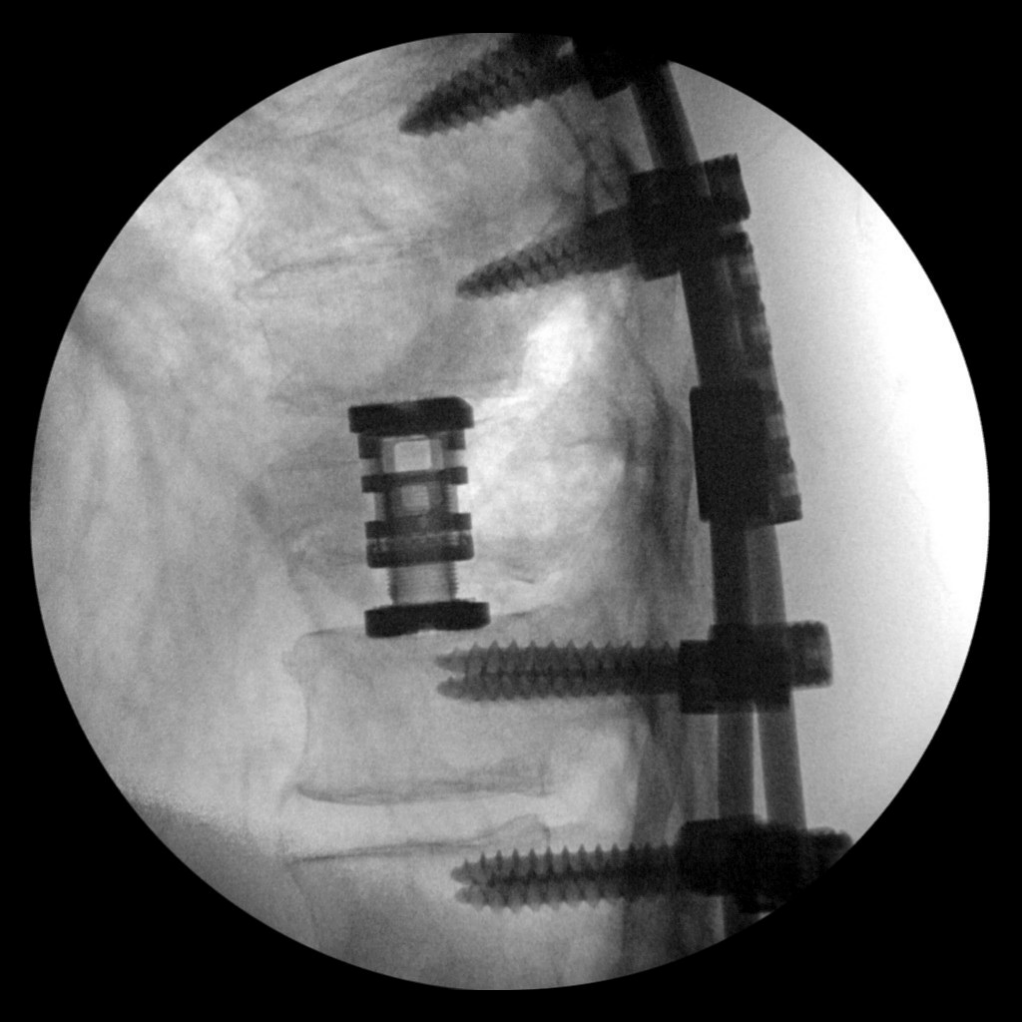
Postoperative lateral radiograph showing placement of T10 cage

A 63-year-old male presented with a T10 burst fracture one level above a previous fusion. Initially, he underwent posterior fixation. Due to continued pain and progressive deformity from the burst fracture shown in Figure 1, he underwent a subsequent minimally invasive lateral T10 corpectomy with osteoplastic rib preservation shown in Figure 2. The patient was discharged home on postoperative day 3.

**Figure 3:**
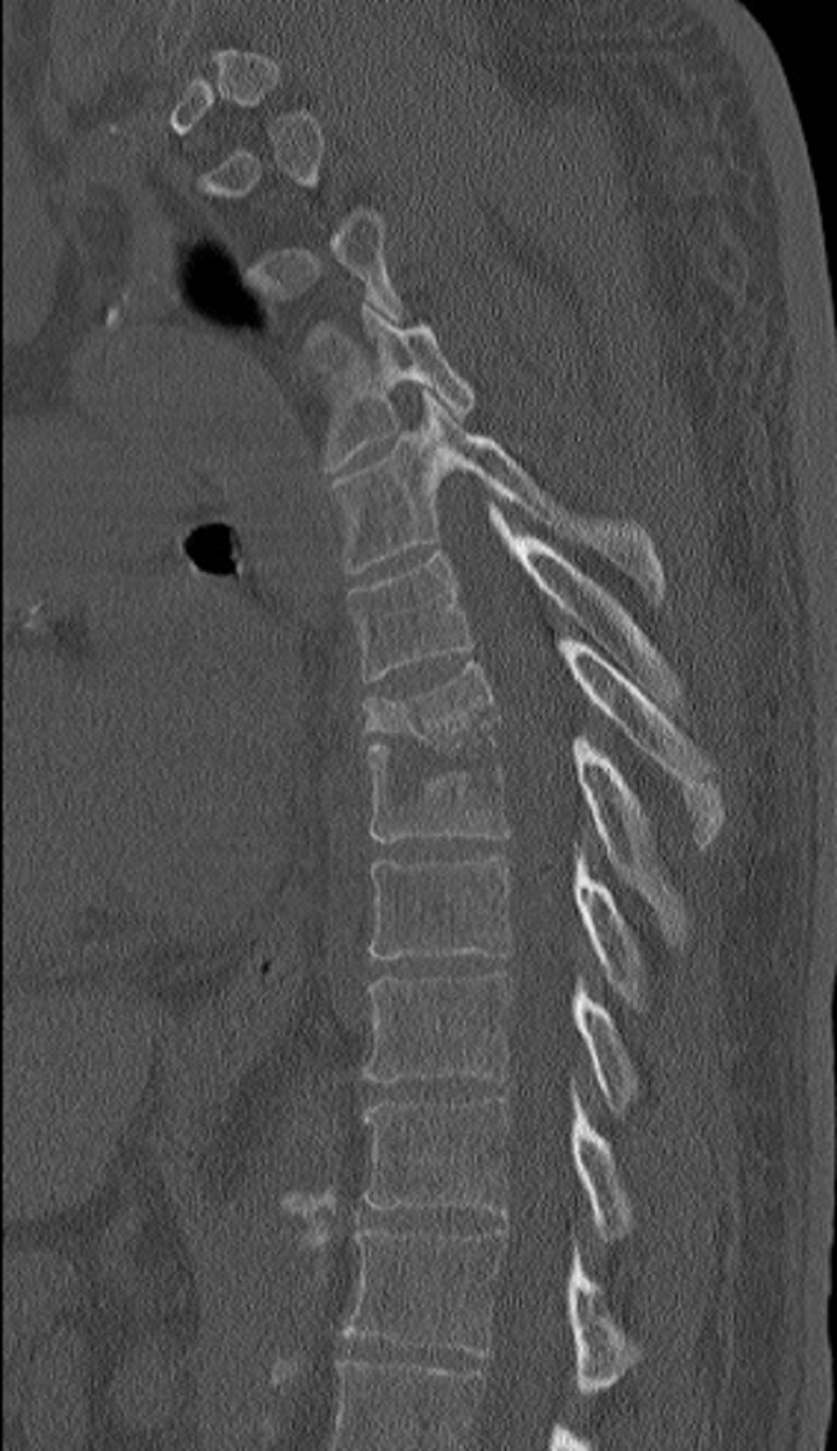
Preoperative sagittal CT demonstrating a T7-8 discitis and osteomyelitis

**Figure 4:**
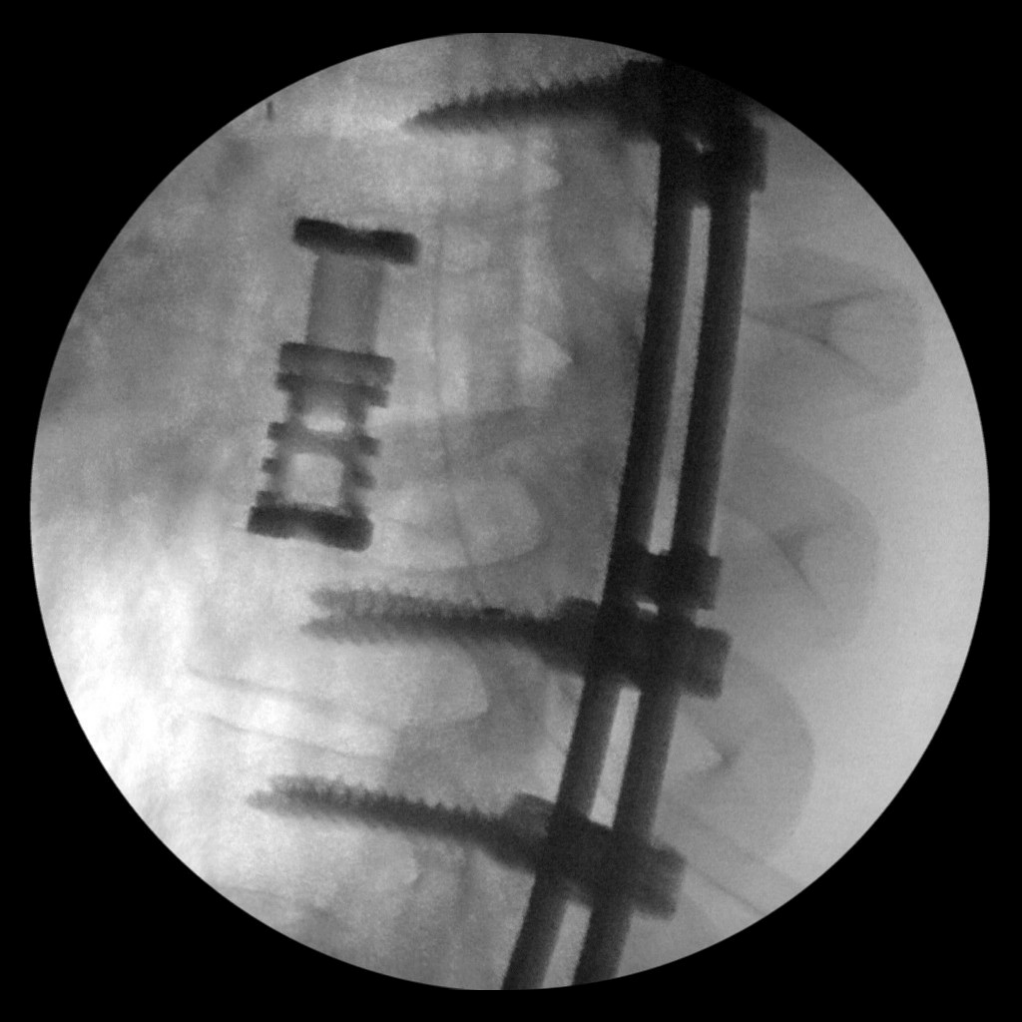
Postoperative lateral radiograph showing placement of T7-8 cage

The second patient was a 51-year-old male with a history of acute T7-8 discitis and osteomyelitis shown in Figure 3. The patient underwent a posterior fixation, followed by a lateral T7-8 corpectomy with an interbody cage using osteoplastic rib preservation as shown in Figure 4. Postoperatively, the patient was able to mobilize with minimal discomfort, transitioning to an oral pain medication regimen the day after surgery. He was discharged home on postoperative day 5 with ongoing systemic antibiotics.

Informed patient consent for both patients was obtained prior to their treatment.

### Operative technique

**Figure 5:**
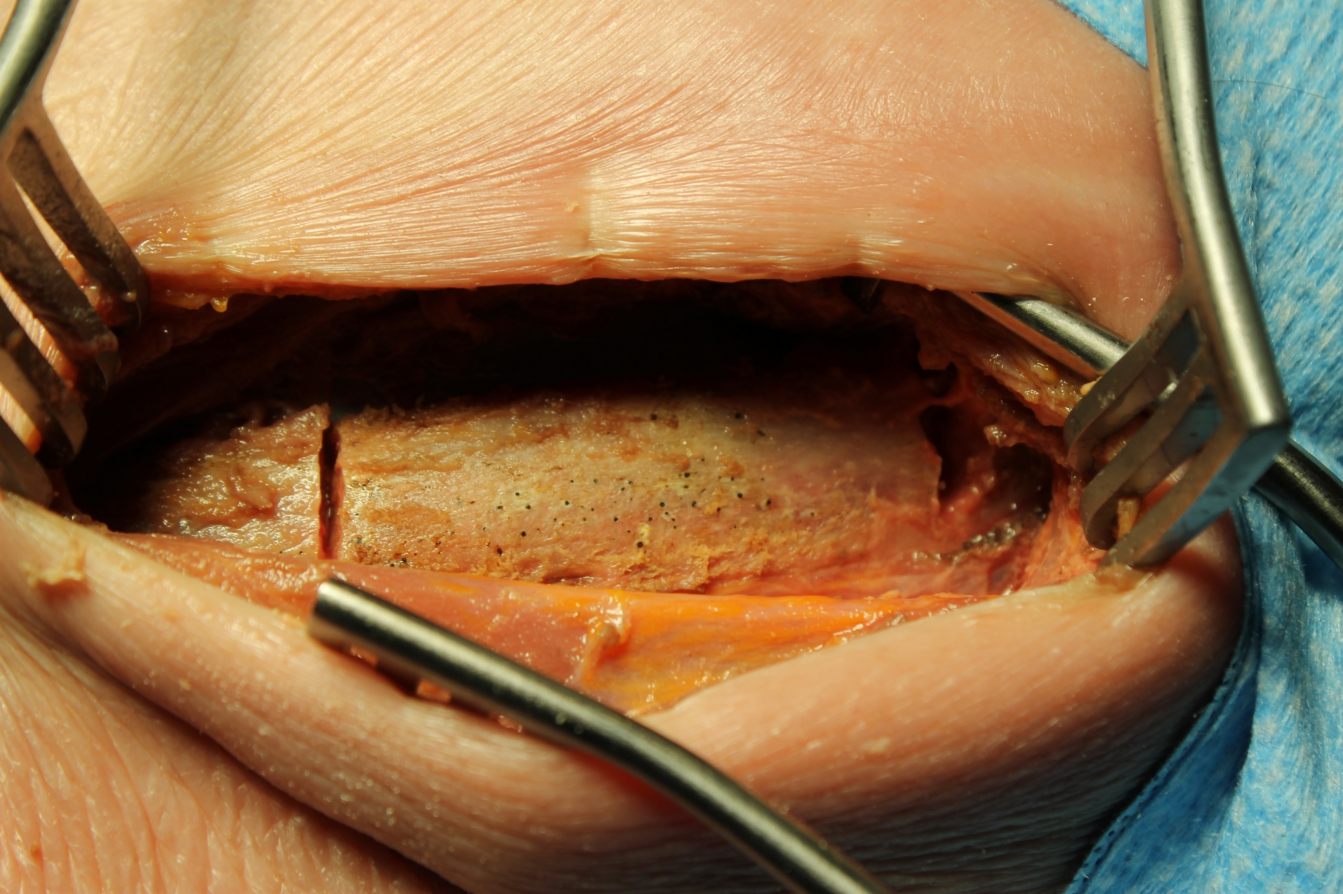
Cadaver specimen showing initial anterior and posterior cuts.

Patients are placed in the lateral decubitus position, the levels are identified, and incision planning is made using standardized techniques [1].  When a rib overlies the planned operative corridor, a decision is made for inferior rib reflection. Initially, an incision is made following the course of the rib. The soft tissue overlying a 3 to 4 cm segment of the rib is dissected away from the rib using a combination of electrocautery with blunt and sharp dissection. The dissection is continued across the anterior portion of the exposed rib in order to release the intercostal muscles. Working from the superior aspect of the rib with the use of curettes and a Doyen dissector, the pleura and intercostal muscles are freed from the rib segment with careful attention not to detach the intercostal neurovascular bundle from the inferior aspect of the rib. The rib segment, now circumferentially cleared of soft tissues, is reflected by making two parallel transverse troughs through the rib at the limits of exposure using a matchstick burr (Midas Rex® Legend tool with an M8 burr match head fluted, 3 mm) (Medtronic, Minneapolis, MN) demonstrated in Figure 5.

**Figure 6:**
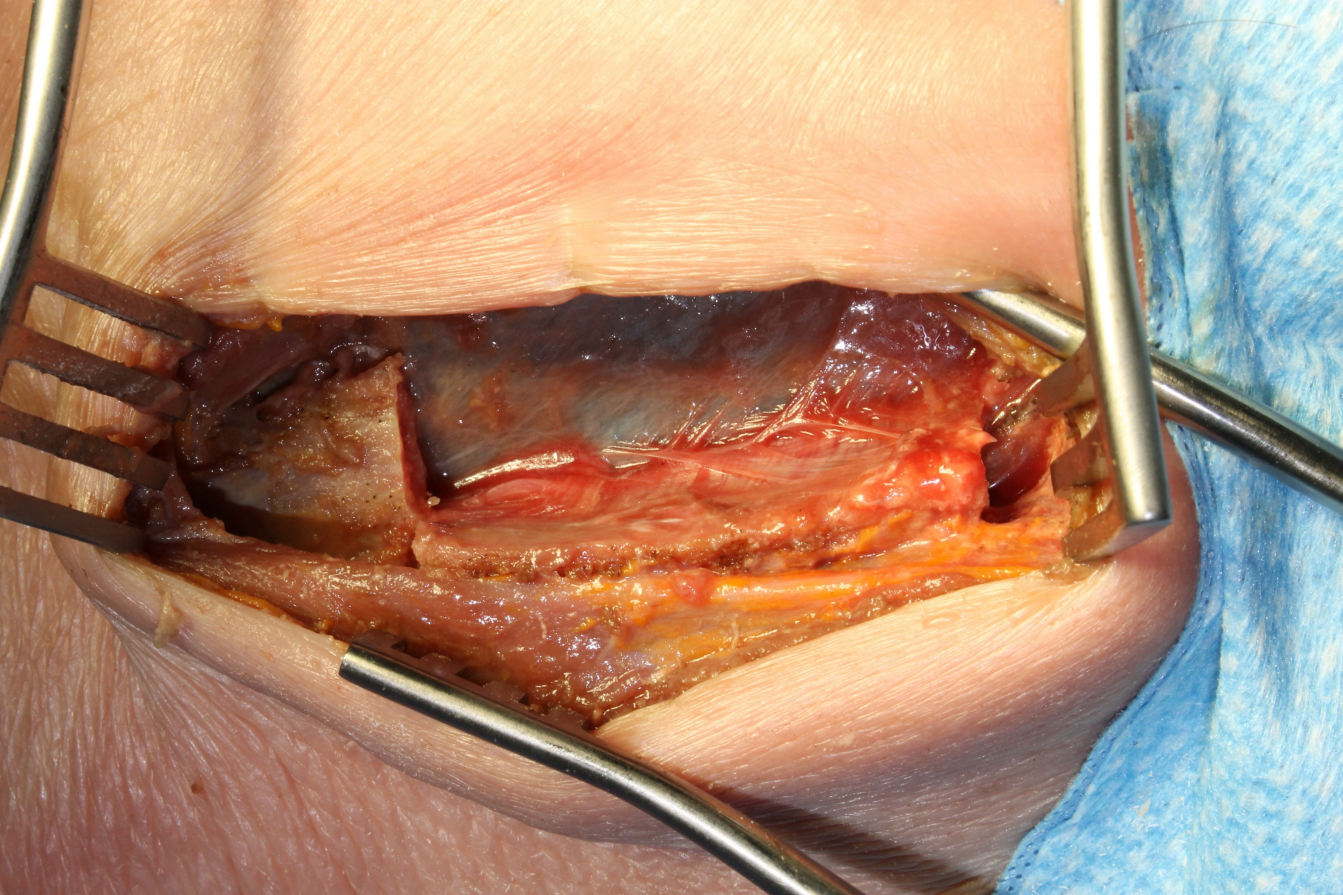
Cadaver specimen showing the rib being reflected inferiorly with its neurovascular bundle

Using a 2 mm Kerrison rongeur, the rib is completely detached and reflected inferiorly with its neurovascular bundle completely preserved as shown in Figure 6.

**Figure 7:**
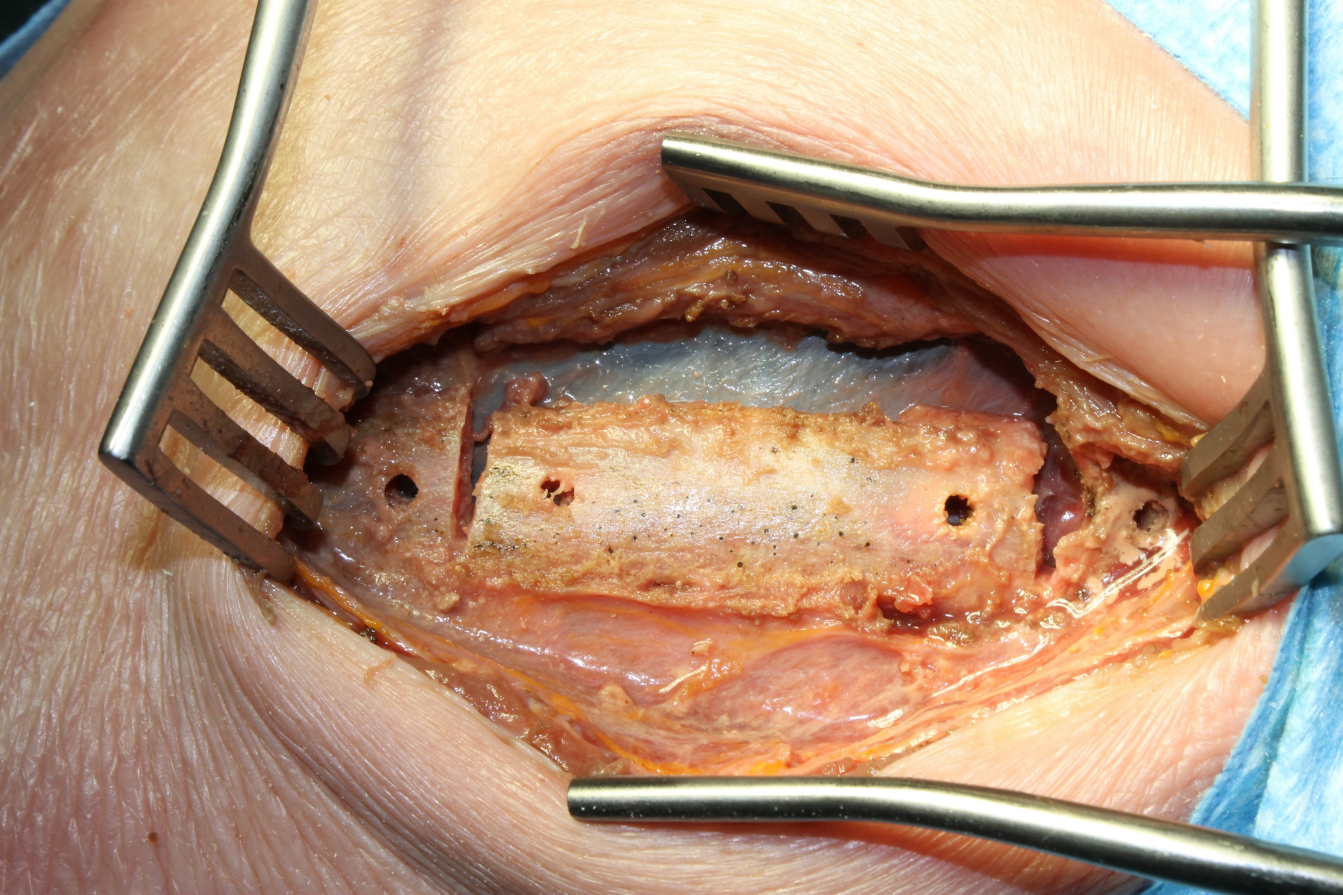
Cadaver specimen showing rib back in its anatomical position with drilled pilot holes.

**Figure 8:**
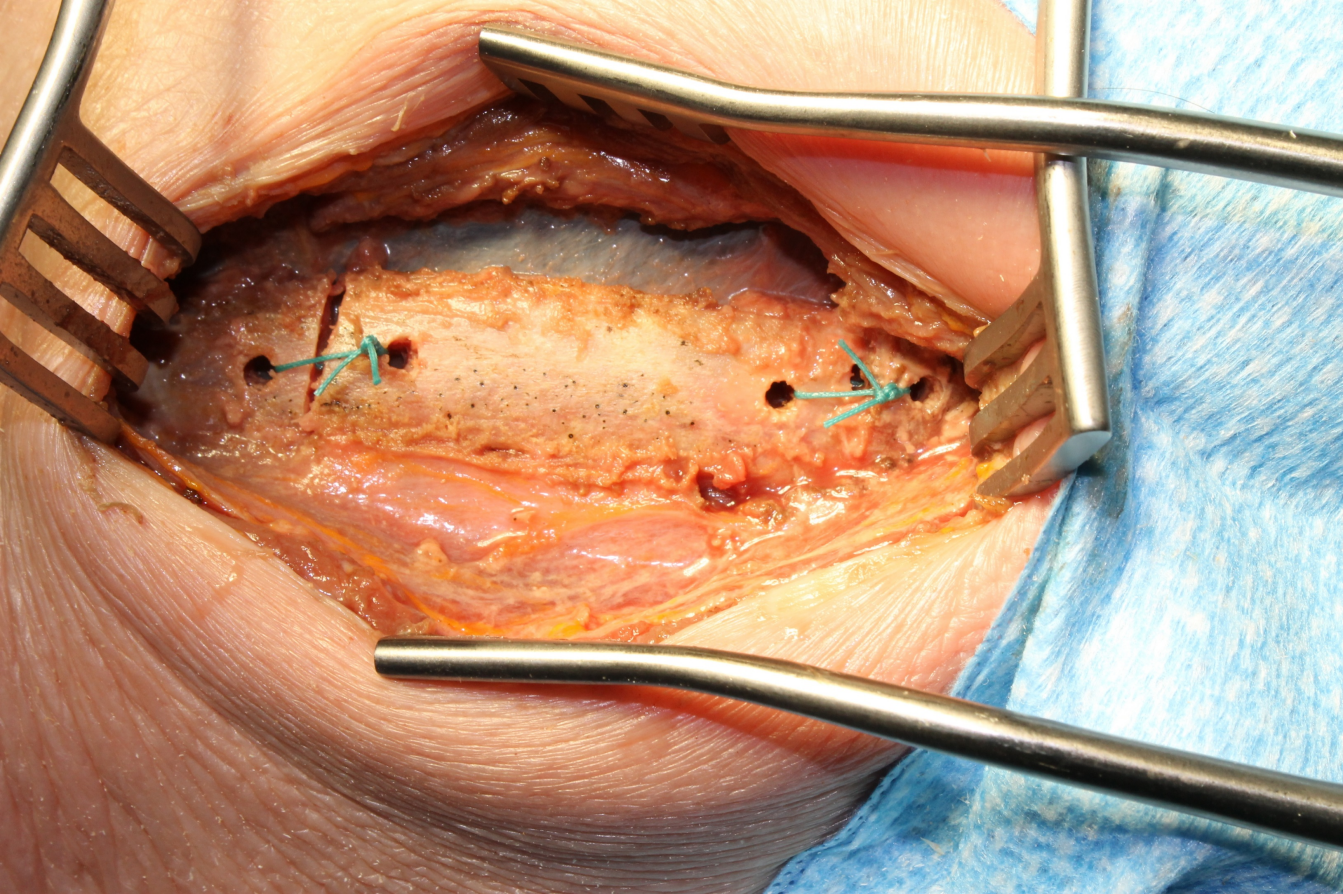
Cadaver specimen showing rib sutured back in its anatomical place.

With unobstructed access to the retropleural space now achieved, the disc spaces and vertebral bodies can be accessed and pathology addressed as previously described [1]. At the conclusion of the case, rather than discarding the rib segment, it is reflected back to its anatomical position and secured with 0-Ethibond non-absorbable sutures (Ethicon, Blue Ash, OH) through pre-drilled holes as shown in Figures 7-8. A small amount of sustained release local anesthetic is placed at the site and then the wound is closed in standard fashion.  

## Discussion

Over a decade ago, the lateral transpsoas retroperitoneal approach was described as a minimally invasive corridor to the lumbar spine [1-2]. This approach has since been further used to access the thoracolumbar junction and lower thoracic spine. Previously, when a rib was encountered, which interfered with the trajectory of the approach, it was resected^ ^and used as autograft [3]. By replacing this rib segment, we believe that patients have experienced less pain resulting in earlier mobilization and decreased the length of hospital stay. 

Pain attributable to a focal flail rib segment as the result of surgical procedures has been described in reports in the cardiothoracic surgery literature, resulting in increased attention to performing chest wall reconstruction [4-7]. Billè, et al. investigated rib fixation after trauma, which demonstrated good long-term results in terms of reducing pain [8]. Additionally, the urological literature describes a subcostal rib-sparing mini donor nephrectomy in order to decrease morbidity and hospital stay [9]. Similarly, the plastic surgery literature has reported rib-sparing techniques to expose the internal mammary vessels in breast reconstruction, again demonstrating reduced postoperative pain [10]. Mayberry, et al. reported a case series of 10 patients with flail chest or chest pain and rib instability who underwent rib fracture repair with absorbable plates and screws. All patients with pain and instability were found to have rapid subjective improvement in their preoperative symptoms [11].

We believe that the rib reconstructive technique reported herein has not been described previously for spinal procedures. This is not surprising as the sacrificed rib segment is often utilized as autograft for interbody fusion. Additionally, our technique differs from rib reconstruction described in reports from the cardiothoracic surgery literature in that we use suture anchoring rather than plating. Initially, in our first case, we used cranial plates for fixation but have since changed to the suture secured (as described in our two case illustrations) method described above with similar and more cost-effective results simply based on our experience. Admittedly, for fusion, autograft is superior to allograft and the decision to reserve the rib segment for osteoplastic reconstruction rather than for fusion has to be considered on a case-by-case basis.  

## Conclusions

We describe an osteoplastic rib-sparing technique for lateral spine surgery. Based on our observations, patients experience less thoracic pain postoperatively, mobilize more quickly, and are discharged earlier than when rib segments are sacrificed.  
